# Effect of Kaolin Geopolymer Ceramics Addition on the Microstructure and Shear Strength of Sn-3.0Ag-0.5Cu Solder Joints during Multiple Reflow

**DOI:** 10.3390/ma15082758

**Published:** 2022-04-08

**Authors:** Nur Syahirah Mohamad Zaimi, Mohd Arif Anuar Mohd Salleh, Mohd Mustafa Al-Bakri Abdullah, Nur Izzati Muhammad Nadzri, Andrei Victor Sandu, Petrica Vizureanu, Mohd Izrul Izwan Ramli, Kazuhiro Nogita, Hideyuki Yasuda, Ioan Gabriel Sandu

**Affiliations:** 1 Faculty of Chemical Engineering Technology, Universiti Malaysia Perlis (UniMAP), Arau 02600, Perlis, Malaysia; syahirahzaimi25@gmail.com (N.S.M.Z.); izzatinadzri@unimap.edu.my (N.I.M.N.); izrulizwan@unimap.edu.my (M.I.I.R.); 2 Center of Excellence Geopolymer & Green Technology (CeGeoGTech), Universiti Malaysia Perlis (UniMAP), Taman Muhibbah, Jejawi, Arau 02600, Perlis, Malaysia; 3 Faculty of Materials Science and Engineering, “Gheorghe Asachi” Technical University of Iasi, 41 “D. Mangeron” Street, 700050 Iasi, Romania; sav@tuiasi.ro (A.V.S.); peviz@tuiasi.ro (P.V.); 4 Romanian Inventors Forum, Str. Sf. P. Movila 3, 700089 Iasi, Romania; 5 Technical Sciences Academy of Romania, Dacia Blvd 26, 030167 Bucharest, Romania; 6 Nihon Superior Centre for the Manufacture of Electronic Materials, School of Mechanical and Mining Engineering, The University of Queensland, Brisbane, QLD 4072, Australia; k.nogita@uq.edu.au; 7 Department of Materials Science and Engineering, Kyoto University, Sakyo-Ku, Kyoto 606-8501, Japan; yasuda.hideyuki.6s@kyoto-u.ac.jp

**Keywords:** multiple reflows, synchrotron, composite solder

## Abstract

Solder interconnection in three-dimensional (3D) electronic packaging is required to undergo multiple reflow cycles of the soldering process. This paper elucidates the effects of multiple reflow cycles on the solder joints of Sn-3.0Ag-0.5Cu (SAC305) lead (Pb)-free solder with the addition of 1.0 wt.% kaolin geopolymer ceramics (KGC). The samples were fabricated using powder metallurgy with the hybrid microwave sintering method. Apart from using conventional cross-sectioned microstructure imaging, advanced synchrotron real-time in situ imaging was used to observe primary IMC formation in SAC305-KGC solder joints subjected to multiple reflow soldering. The addition of KGC particles in SAC305 suppressed the Cu_6_Sn_5_ IMC’s growth as primary and interfacial layers, improving the shear strength after multiple reflow soldering. The growth rate constant for the interfacial Cu_6_Sn_5_ IMC was also calculated in this study. The average growth rate of the primary Cu_6_Sn_5_ IMCs decreased from 49 µm/s in SAC305 to 38 µm/s with the addition of KGC particles. As a result, the average solidified length in the SAC305-KGC is shorter than SAC305 for multiple reflow soldering. It was also observed that with KGC additions, the growth direction of the primary Cu_6_Sn_5_ IMC in SAC305 changed from one growth to two growth directions. The observed results can be attributed to the presence of KGC particles both at grains of interfacial Cu_6_Sn_5_ IMCs and at the surface of primary Cu_6_Sn_5_ IMC.

## 1. Introduction

Solders play a crucial role in electronic packaging via their provision of mechanical support and continuous electrical connection between the substrates and electronic components. Reflow soldering has been commonly used to form solder interconnection at the component and board-level assemblies. The emergence of complex electronic packagings such as System-in-Package (SiP) and Package-on-Package (POP) require multiple reflow soldering to form all the solder interconnections. In advanced electronic packaging, additional solder rework is required [1], and this process will result in interconnections undergoing more than one reflow cycle. Intermetallic compounds (IMC) will be formed during the reactions between Cu substrates and Sn solder alloys during the soldering process [2,3]. The formation of IMC is crucial as it influences the reliability of the solder joints. It is also inevitable that the thickness and morphology of the IMC layer will grow and evolve with increasing time and temperature [4,5]. Therefore, in the case of multiple reflow cycle processes, the solder joints in the first reflow cycle will undergo further changes in the thickness and morphology of the IMC layer during the subsequent reflow cycles. Researchers reported that the thickness of the IMC layer would increase during the multiple reflow soldering process, affecting the solder joint’s reliability [6,7,8]. Additionally, a thicker formation of the IMC layer could result in a brittle fracture, which would degrade the strength of the solder joints [9,10,11,12]. The IMCs such as Cu_6_Sn_5_ and Ag_3_Sn are inherently brittle in nature. It consists of one or more covalent compounds and will be deformed in a brittle manner under mechanical loads [13]. Thus, with a thicker layer of IMC, it can increase the likelihood of failure in the solder joints [14]. Sn–Ag–Cu (SAC) solder alloy is commonly used in the electronics industry [15,16] and is touted as a viable substitute for Sn–Pb solder alloy due to its low melting point. Moreover, the Solder Value Product Council (SPVC) has approved SAC solder alloy as one of the Pb-free solder alloys that can replace Sn–Pb solder alloy [12]. However, a significant concern in using the SAC solder alloy is that the IMC layer’s growth is faster than Sn–Pb solders due to the higher service temperature in the SAC solder [9]. Therefore, controlling growth on the formation of the IMC layer in SAC solder alloy, especially during the multiple reflow soldering process, is vitally important to preserve the reliability of the solder joints.

As the quality and reliability of solder joints are dependent on the formation of IMC layers, many researchers took the initiative to enhance the performance of existing Pb-free solder alloys. One of the feasible and viable approaches was using ceramics materials to form composite solders [17,18,19,20,21]. To date, there are various ceramic materials that had been successfully added into the solder matrix, such as silicon carbide (SiC) [12], titanium oxide (TiO_2_) [17,18,22], titanium carbide (TiC) [22], samarium oxide (Sm_2_O_3_) [23], alumina (Al_2_O_3_) [24], and cerium oxide (CeO_2_) [25,26]. These ceramic particles did not react with the phase of the solder matrix, thus forming no new compounds within the solder during the melting process [27]. The added ceramic particles also functioned as a second-strengthening phase in the solder matrix, as their properties remain intact within the solder matrix, which strengthens the solder alloys [27]. The dispersion of ceramic particles in the solder matrix increases nucleation rates that result in grain refinement [28]. Tang et al. [29] reported that the grain size of Cu_6_Sn_5_ IMC increased with increasing reflow time, but the addition of TiO_2_ suppressed the growth of the IMC due to TiO_2_ particles preventing the diffusion between Cu and Sn atoms. It was also reported that the solder properties improved due to the enhancement in the growth of the interfacial IMC layer [20,24,26,30]. Therefore, it can be surmised that the growth of the IMC layers during multiple reflow soldering needs to be controlled or limited as it improves solder properties and ensures the reliability of the solder joints.

Geopolymers are inorganic polymers that are formed through the geopolymerization process [31].The process of geopolymerization occurs as the aluminosilicate sources, which consist of SiO_2_ and Al_2_O_3_ are dissolute in a highly alkaline activated solution. The geopolymerization process results in the formation of a semi-crystalline structure with Si–O–Al and Si–O–Si bonds. The geopolymers are advantageous as they can transform to a crystalline structure using slightly low sintering temperature during the sintering process with excellent mechanical properties compared to typical ceramics. The fabrication of the geopolymer ceramics seems to be advantageous as it is energy efficient. Moreover, geopolymer ceramics consists of several elements such as Si and Al which may also contribute to the properties of the solder alloy. In our previous research [30,32] the effect of the addition of kaolin geopolymer ceramic (KGC) onto the properties of Sn-3.0Ag-0.5Cu (SAC305) under as-reflowed and isothermal aging conditions was investigated. The results confirmed that the addition of KGC as reinforcement particles in SAC305 enhanced its properties in as-reflowed and isothermal aging conditions. Furthermore, the segregation of KGC in the SAC305 matrix refined the microstructure. It suppressed excessive growth of the interfacial IMC even in high isothermal aging temperatures for more extended periods, which improved solder properties such as solder joint strength and the solderability of SAC305. There are many works in the literature focussing on the interfacial reactions between Sn–Cu-based alloys and substrates during multiple reflow soldering [8,33,34]. As reported by S.Tikale et al. [24], the addition of Al_2_O_3_ effectively suppressed the growth of Cu_6_Sn_5_ IMC [24]. Owing to the ability of Al_2_O_3_ particles to hinder the diffusion of Cu to the liquid solder, results in suppression of the IMC layer under multiple reflow soldering [24]. M.A.A Mohd Salleh et al. [1] discovered that the addition of TiO_2_ suppressed Cu_6_Sn_5_ IMC both as primary and interfacial during multiple reflow soldering and thus can improve the shear strength of solder. Nevertheless, limited studies have been reported on the behavior of primary IMC in the solder alloys with the addition of reinforcement particles during multiple cycles of reflow soldering [1]. Primary IMC such as Cu_6_Sn_5_ and Ag_3_Sn are relatively brittle in the solder bulk [35,36]. Therefore, their behavior and distributions in the bulk solder, specifically during multiple reflow soldering, significantly influence the reliability of the joints. This paper also analyses the formation of IMC Cu_6_Sn_5_ as primary crystals and interfacial layers in the solder during multiple reflow soldering using advanced techniques such as in situ synchrotron X-ray imaging.

## 2. Materials and Methods

### 2.1. Materials

**Figure 1:**
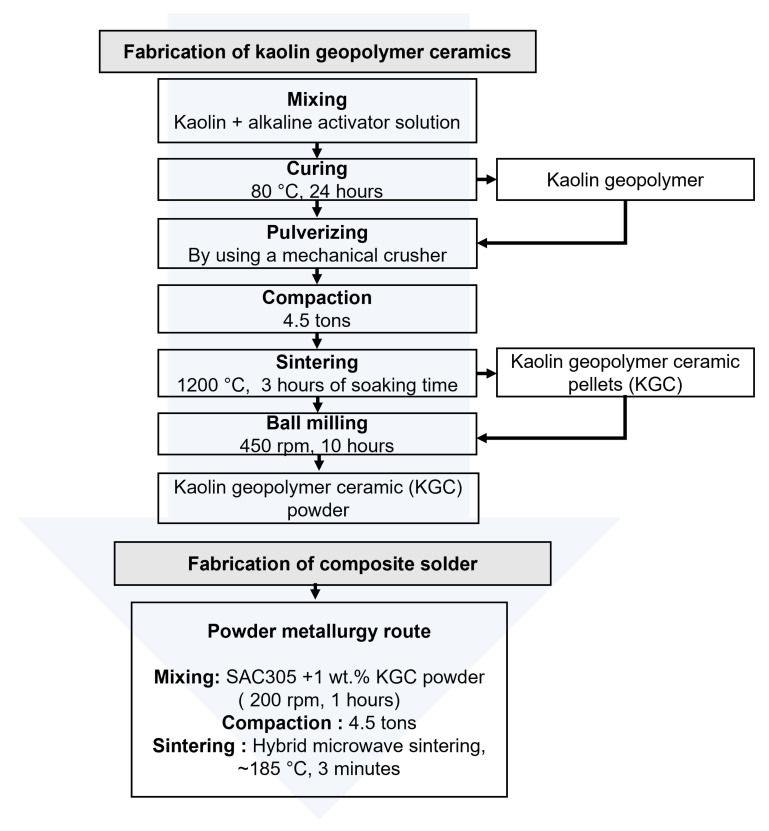
Fabrication process of kaolin geopolymer ceramics and composite solder.

Figure 1 shows the flowchart of the kaolin geopolymer ceramic and composite solder fabrication process. Sn-3.0Ag-0.5Cu (SAC305) powders were used as the solder matrix material. It has a spherical morphology and an average size of ~25–45 µm, purchased from Nihon Superior Co. Ltd. (Osaka, Japan). The kaolin geopolymer ceramic (KGC) powders with an average particle size of ~18 µm were used as the reinforcement material.

### 2.2. Fabrication of Kaolin Geopolymer Ceramic

The kaolin geopolymer ceramic (KGC) fabrication began with the formation of kaolin geopolymer via the geopolymerization process. Kaolin was purchased from Associated Kaolin Industries Sdn. Bhd. and used as the raw material to produce KGC. The kaolin was geopolymerized using an alkaline activator solution, then cured in an oven at 80 °C for 24 h to produce kaolin geopolymer. Next, the product was crushed using a mechanical crusher and compacted at a load of 4.5 tons. The compacted pellets were sintered at 1200 °C at 3 h of soaking time to produce KGC. Then, the KGC pellets were ball-milled for 10 h in a planetary mill at a speed of 450 rpm with a ball to powder ratio of 10:1 to produce KGC particles with an average size of ~18 µm.

### 2.3. Fabrication of Composite Solder

A composite solder was developed by reinforcing 1 wt.% of kaolin geopolymer ceramic (KGC) with SAC305 solder powder. The composite solder was fabricated using powder metallurgy with a hybrid microwave sintering method. The SAC305 solder powder and 1 wt.% KGC were weighed, then the mixture was mixed in an airtight container using a planetary mill machine at 200 rpm. The product was uniaxially compacted at a load of 4.5 tons. The spherical compacted pellets were then sintered using the hybrid microwave sintering method at ~185 °C under ambient conditions for ~3 min in a 50 Hz microwave oven. A microwave susceptor material of SiC was used for sintering. A sample of SAC305 without the addition of KGC particles was also fabricated using the same approach.

### 2.4. Microstructure Analysis

The microstructure of the SAC305 and SAC305-KGC was analyzed using a scanning electron microscope (SEM). A sintered pellet was cold-rolled using a rolling machine until the thickness of the sheets was ~50 µm to produce a solder ball. Then, the sheets were punched using a 3.0 mm metal puncher, dipped with rosin mildly activated flux, and reflowed on a Pyrex glass to produce a solder ball with a diameter of ~900 µm. The solder balls were sieved to standardize their average size, then reflowed on a Cu substrate printed circuit board (PCB) with an organic solderability preservative (OSP) using an F4N desktop reflow oven. A small amount of rosin mildly activated flux was applied onto the sample’s surface. The flux helped eliminate any contaminations and oxidation before and during the melting process. After that, the reflowed samples SAC305 and SAC305-KGC were cross-sectioned, cold mounted, and grounded with a different grit size of SiC papers. The samples were polished using alumina and colloidal silica suspension to obtain a clearer image of the microstructure under SEM. The average thickness of the IMC layer was measured on the cross-sectioned samples using ImageJ. The IMC thickness (*x*) was calculated according to Equation (1). The 3D primary intermetallic in the solder joint was microstructurally analyzed as well, where it was etched using an etchant solution from a mixture of 2% 2-nitrophenol, 5% sodium hydroxide, and 93% distilled water to prepare it for analyses.

*x* = *A*/*L* (1)

where *x* is IMC thickness, *A* is the area of the IMC layer and *L* is the length of the IMC layer.

### 2.5. In Situ Synchrotron X-ray Radiography Imaging

**Figure 2:**
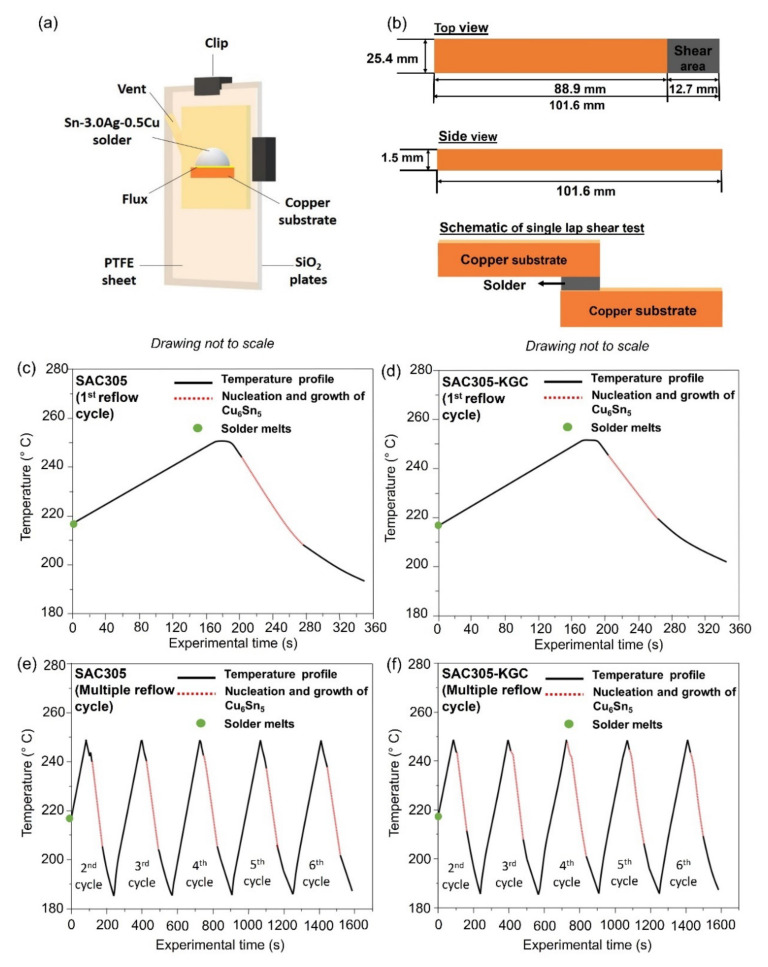
(**a**) Schematic diagram of sample cell for the in situ soldering synchrotron observation, (**b**) Schematic diagram illustrating the configuration for single lap shear test, temperature profile, and growth behavior of primary Cu_6_Sn_5_ for (**c**) SAC305 1st reflow cycle, (**d**) SAC305-KGC 1st reflow cycle, (**e**) SAC305 multiple reflow cycle (2nd to 6th cycle), and (**f**) SAC305-KGC multiple reflow cycle (2nd to 6th cycle).

In situ synchrotron X-ray radiography imaging was conducted using beamline BL20XU at Spring-8 synchrotron in Hyogo, Japan. The experiment was conducted according to the solidification observation setup developed and reported in [1,37,38]. In the experiment, thin sheets of SAC305 and SAC305-KGC were aligned vertically on the 100 µm thick copper (Cu) printed circuit board (PCB), and a small amount of flux was applied. Then, the samples were sandwiched between two glass plates of silica, SiO_2_, and polytetrafluoroethylene (PTFE) spacer sheets with an observation window area of 10 × 10 mm^2^, as depicted in Figure 2a. The PTFE sheets were also cut to form vents for flux outgassing purposes during the soldering process. To mimic the soldering process, a furnace equipped with graphite heating elements was used, and the reflow profile JEDEC standard (JESD22-A113D) was used. During the soldering process, the samples were heated from room temperature to 250 °C at a rate of 0.33 °C/s, held for 30 s at the peak temperature, before being cooled at 0.33 °C/s for 6 cycles as in Figure 2c–f. The X-ray energy used was 21 keV. A planar undulator was used, acting as a light source, and the radiations produced were then monochromatized using Si double crystal monochromators. The image detector located at ~2.5–3.0 m away from the samples collected the image signals, which were converted into a digital format of 2000 × 2000 pixels, resulting in a resolution of 0.47 µm/pixel and a viewing field of 1 mm × 1 mm. The parameters used in this experiment were selected to provide a high degree of coherence, absorption, and phase contrast, allowing the boundaries of the samples to be observed in the transmitted images.

### 2.6. Single Lap Shear Testing

Solder joint strength after multiple reflow cycles was evaluated using a single lap shear test, performed using an Instron Machine. The specifications of the copper substrate (PCB-FR4 type) followed the ASTM D1002 standard, as shown in Figure 2b. The fractography of the solder joint after the test was imaged using a scanning electron microscope (SEM) equipped with energy-dispersive X-ray spectroscopy (EDS) under secondary imaging mode to investigate the possible fracture surface mechanism after shearing loads.

## 3. Results and Discussions

### 3.1. Microstructure Analysis

#### 3.1.1. Ex Situ Microstructure Analysis of Solder Joints after Multiple Reflows

**Figure 3:**
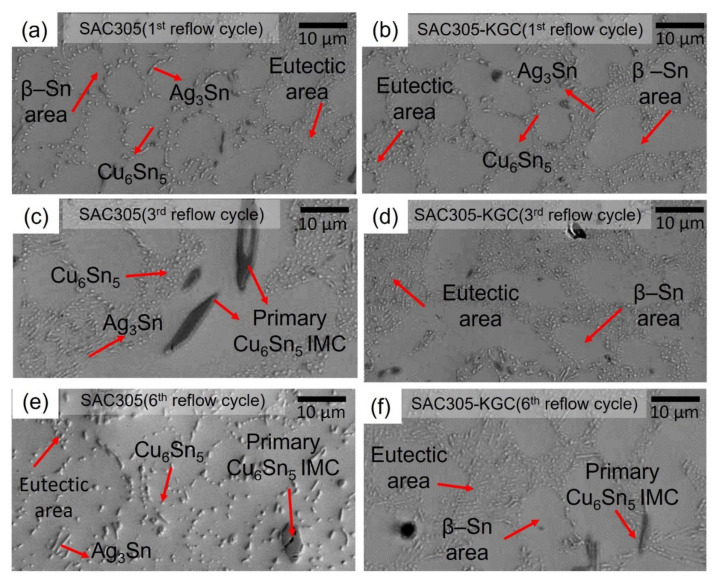
Cross-sectioned microstructure at the bulk solder joints (**a**) SAC305 at 1st reflow cycle, (**b**) SAC305-KGC at 1st reflow cycle, (**c**) SAC305 at 3rd reflow cycle, (**d**) SAC305-KGC at 3rd reflow cycle, (**e**) SAC305 at 6th reflow cycle, and (**f**) SAC305-KGC at 6th reflow cycle.

The microstructure of the solidified SAC305 and SAC305-KGC solder joints after the first, third, and sixth cycles of reflow soldering is shown in Figure 3. The microstructure of SAC305 solder alloys consists of fractions of β-Sn phase and eutectic phases. Based on Figure 3, the β-Sn and eutectic areas were observed in both materials of SAC305 and SAC305-KGC solder joints for multiple cycles of reflow soldering. Fine dots Cu_6_Sn_5_ and needle-like Ag_3_Sn IMC formed in the eutectic area as observed in Figure 3. In this study, hypoeutectic SAC305 solder was soldered on a copper substrate and as a result of Cu diffusion and dissolution during soldering, primary Cu_6_Sn_5_ will form in the solder joint [26,33]. Per Figure 3a,c,e, IMC particles in the eutectic areas of the SAC305 solder joints are coarse compared to the SAC305-KGC solder, suggesting that the addition of kaolin geopolymer ceramic (KGC) in SAC305 solder alloy suppresses further coarsening of IMCs in the eutectic area after multiple cycles of reflow soldering.

**Figure 4:**
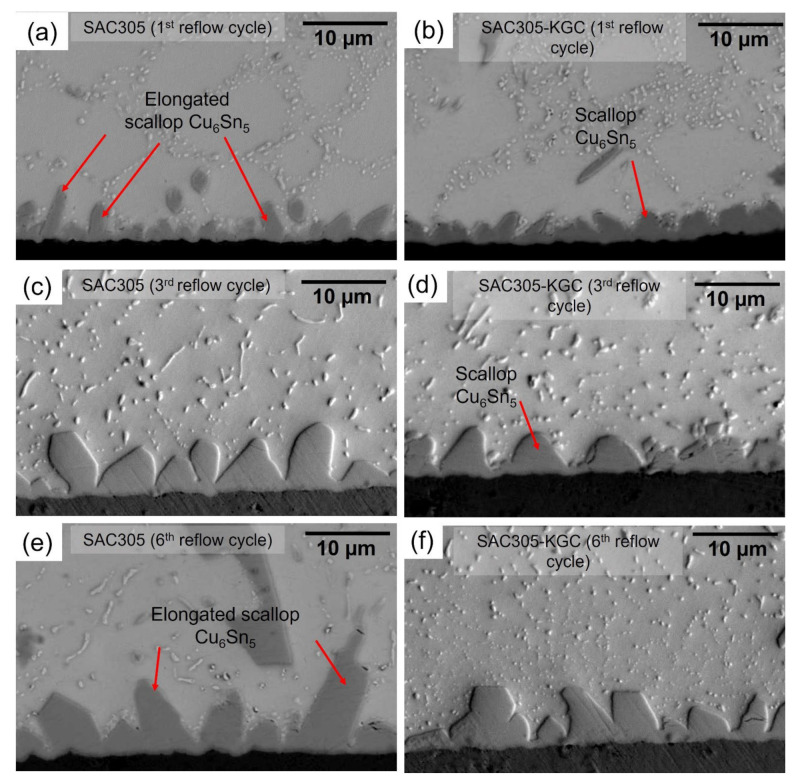
Cross-sectioned microstructure at the interfacial of solder joints (**a**) SAC305 at 1st reflow cycle, (**b**) SAC305-KGC at 1st reflow cycle, (**c**) SAC305 at 3rd reflow cycle, (**d**) SAC305-KGC at 3rd reflow cycle, (**e**) SAC305 at 6th reflow cycle, and (**f**) SAC305-KGC at 6th reflow cycle.

**Figure 5:**
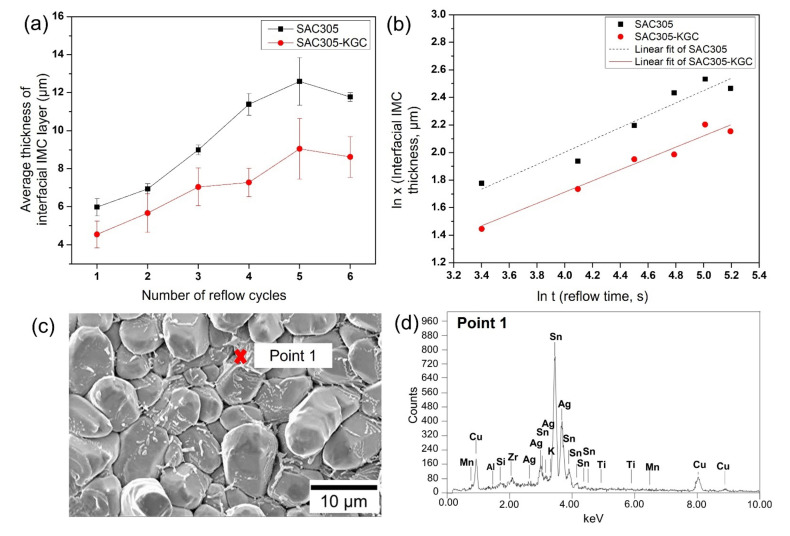
(**a**) Average thickness of interfacial intermetallic compound (IMC) layer at different reflow cycles, (**b**) ln plot growth of interfacial IMC layer respected to different reflow cycles, (**c**) Top view of interfacial IMC layer in SAC305-KGC after 6th cycle reflow, and (**d**) EDX point analysis at Point 1.

During the soldering process, the interfacial reaction between molten solder alloy and copper substrate will form an interfacial intermetallic compound (IMC) layer. The cross-sectional images of solder joints were analyzed, and the thickness of the IMC layer was measured per Figure 4 and Figure 5 to elucidate the effects of KGC addition on the interfacial IMC layer for multiple cycles of reflow soldering. The elongated scallop of Cu_6_Sn_5_ in the SAC305 solder joints was observed to form after reflow soldering, as shown in Figure 4a, suggesting increased concentrations of copper atoms from the substrates to the Sn matrix [26,33]. In the SAC305-KGC solder joints, the small and scalloped shape was formed after reflow soldering; however, with an increasing reflow cycle, the elongated scallop in the SAC305 solder joints became coarser and grew into the solder matrix, as observed in Figure 4c,e. The formation of the elongated scallop IMC layer in SAC305 solder joints is unfavorable, as it could compromise the reliability of the joints by inducing crack formation [32]. However, this trend was not observed in the solder joints of SAC305-KGC since the small and scalloped IMC layer was shorter and became more faceted after multiple cycles of reflow soldering, as can be seen in Figure 4d,f.

The measured average thickness of the interfacial IMC layer for different reflow cycles was plotted and shown in Figure 5a. Initially, the interfacial IMC layer in the SAC305 solder joints grows to an average thickness of ~5.9 µm. After multiple cycles of reflow soldering, the interfacial IMC layer grows to a maximum of ~12.6 µm in SAC305 solder joints. Meanwhile, in the SAC305-KGC solder joints, the interfacial IMC layer grows to an initial average thickness of ~4.5 µm and a maximum of ~9.4 µm after multiple cycles of reflow soldering. The average thickness of the SAC305-KGC solder joints was thinner than SAC305 solder joints, inferring that the addition of KGC might play a role in suppressing the increasing thickness of the IMC layer during multiple cycles of reflow soldering. The thickness of the interfacial IMC layer after multiple cycles of reflow soldering can be generally described per the empirical power law equation [1,6,36,39]:

*x* = *kt*^*n*^ (2)

where *x* is the thickness of the IMC layer at reaction time *t*, *k* is the growth rate constant, and *n* is the time exponent. In this study, the reaction time was based on the time above 250 °C, which is 30 s at each reflow cycle.

According to Liu et al. [40], the interfacial IMC layer’s growth could either be controlled by the grain boundary diffusion at the interface, bulk diffusion, or chemical reaction with the values of time exponent, n, of 0.33, 0.50, or 1.0, respectively. In this study, the values of k and n for SAC305 and SAC305-KGC for multiple cycles of reflow soldering can be obtained by the linear fitting method of the ln-ln graph. Figure 5b shows the graph of linear fitting obtained from the experimental data. The results revealed that the time exponent, n, for the growth of interfacial IMC layer in SAC305 and SAC305-KGC solder were 0.45 and 0.41, respectively. The values obtained were near 0.5, which explains the growth of the interfacial IMC layer during multiple cycles of reflow soldering as controlled via bulk diffusion. Regarding the growth rate constant (k), the interfacial IMC in SAC305-KGC has a k value of 0.37 µm^2^/s, compared to SAC305, which is 0.81 µm^2^/s for multiple reflow cycles. This proved that the growth of the interfacial IMC layer in SAC305 is faster than SAC305-KGC, thus leading to a thicker formation of the interfacial IMC layer after multiple cycles of reflow soldering. Salleh et al. [1] also reported that the addition of TiO_2_ in Sn-0.7Cu solder resulted in the growth exponent of 0.5 with t^1/2^ dependence. A top view with high magnification images of the interfacial IMC layer in the SAC305-KGC after the sixth cycle of reflow soldering is shown in Figure 5c. EDX point analysis was performed on the grains of Cu_6_Sn_5_ IMC_._ The results from the EDX point analysis at “Point 1”, per Figure 5d, confirmed the presence of KGC particles on the surface of Cu_6_Sn_5_ IMC grains. This observation suggests that KGC particles remained in contact with Cu_6_Sn_5_ IMC grains after the sixth cycle of reflow soldering, thus suppressing the growth of interfacial Cu_6_Sn_5_ IMC layer during multiple reflow soldering processes. As mentioned in [34], the channels between the Cu_6_Sn_5_ scallops provide a path for the rapid diffusion and dissolution of copper atoms from the substrates to the molten solder, resulting in the formation of the Cu_6_Sn_6_ interfacial layer. As the growth rate constant calculated in SAC305 was faster than SAC305-KGC, this explains that the rapid diffusion of copper atoms during the solid–liquid process could result in a thicker interfacial Cu_6_Sn_5_ IMC layer with an elongated scalloped shape. Meanwhile, the growth rate constant of the SAC305-KGC solder joints was lower since the presence of the KGC particles on the surface of Cu_6_Sn_5_ grains might block channels between the Cu_6_Sn_5_ scallop for rapid diffusion of copper atoms from the substrate and tin atoms from the molten solder. This explains the thinner interfacial IMC layer in the SAC305-KGC solder joints during the stipulated multiple reflow soldering. Moreover, Tang et al. [9] suggested that the theory of adsorption of surface-active materials can be used to determine the role of the reinforcement particles on the interfacial IMC layer and the IMCs at the bulk solder.

#### 3.1.2. In Situ Observation on Primary Cu_6_Sn_5_ IMC during Multiple Reflows

**Figure 6:**
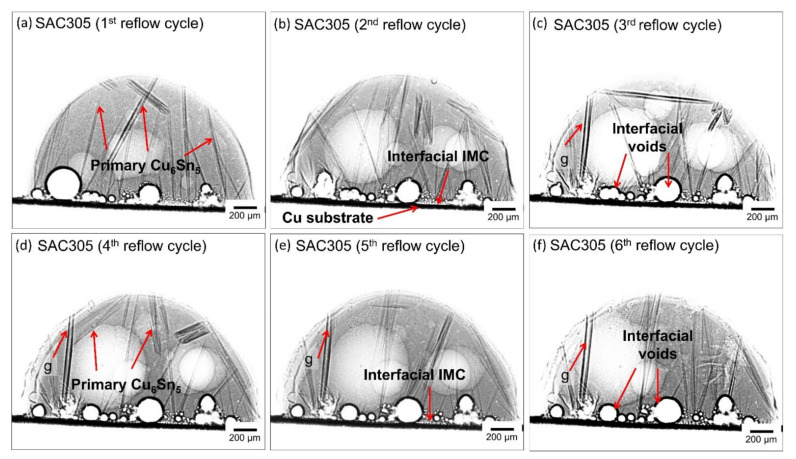
Synchrotron radiation images of SAC305 showing the formation of primary Cu_6_Sn_5_ IMC for multiple cycles of reflow soldering.

**Figure 7:**
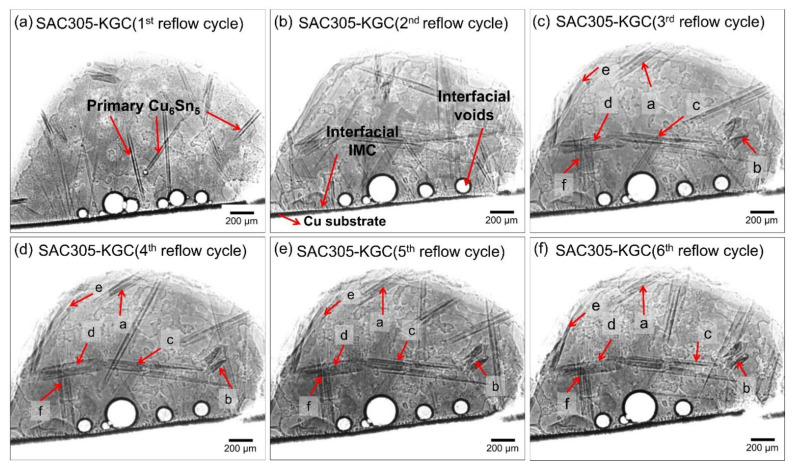
Synchrotron radiation images of SAC305-KGC showing the formation of primary Cu_6_Sn_5_ IMC with respect to multiple cycles of reflow soldering.

**Figure 8:**
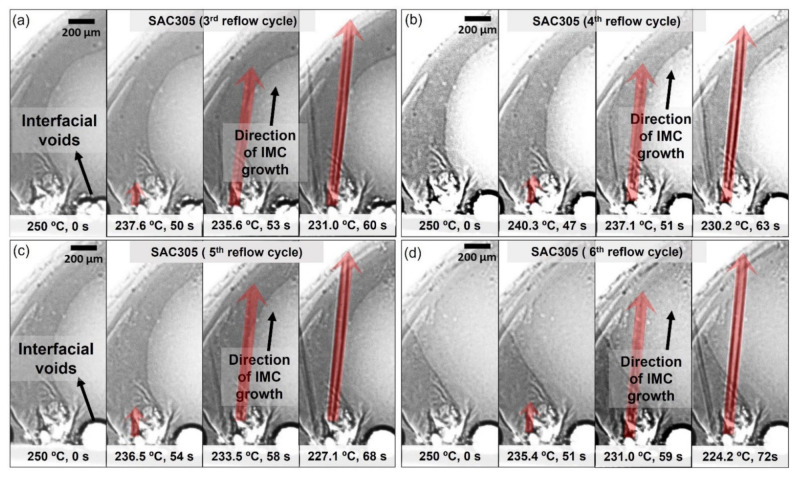
Sequences of the image showing the growth of IMC (denoted by ‘g’) in SAC305 solder joints during (**a**) 3rd reflow cycle, (**b**) 4th reflow cycle, (**c**) 5th reflow cycle, and (**d**) 6th reflow cycle (t = 0 s when the sample begins to cool).

The growth behavior of primary Cu_6_Sn_5_ during multiple reflow soldering in SAC305 and SAC305-KGC solder joints was in situ visualized using synchrotron X-ray imaging at Spring8, Japan. Figure 6 and Figure 7 show the synchrotron radiation images for both SAC305 and SAC305-KGC during multiple reflow soldering. The darker rods in the images are primary Cu_6_Sn_5,_ and a slightly brighter is the Sn liquid. Both figures showed the distribution of primary Cu_6_Sn_5_ formed in the SAC305 and SAC305-KGC solder joints for multiple cycles of reflow soldering. In this experiment, SAC305 and SAC305-KGC solder started to melt at ~217 °C (t = 0 when the solder melts). After a peak temperature of 250 °C for 30 s, the solders began cooling down. During the cooling process for the first reflow cycle of SAC305, the primary Cu_6_Sn_5_ IMC nucleated at experimental times of 202 s to 272 s and temperatures of ~244 °C to 210 °C as in Figure 2c. Meanwhile, in SAC305-KGC (Figure 2d), the primary Cu_6_Sn_5_ IMC starts to nucleate at experimental times of 203 s to 262 s and temperatures of ~245 °C to 220 °C. This implies that during the first cycle of reflow soldering in SAC305-KGC, the primary Cu_6_Sn_5_ IMC took a shorter time to grow from the first IMC nucleation until the completion of the solidification process. The shorter time is taken for the primary Cu_6_Sn_5_ IMC in the SAC305-KGC to grow suggested the occurrence of rapid solidification, which affects the nucleation growth time with inhibits the tip growth of primary IMC [41]. Besides that, there are also a number of interfacial voids as in Figure 6, Figure 7 and Figure 8. The formation of interfacial voids was caused by flux outgassing during soldering [37,42].

**Figure 9:**
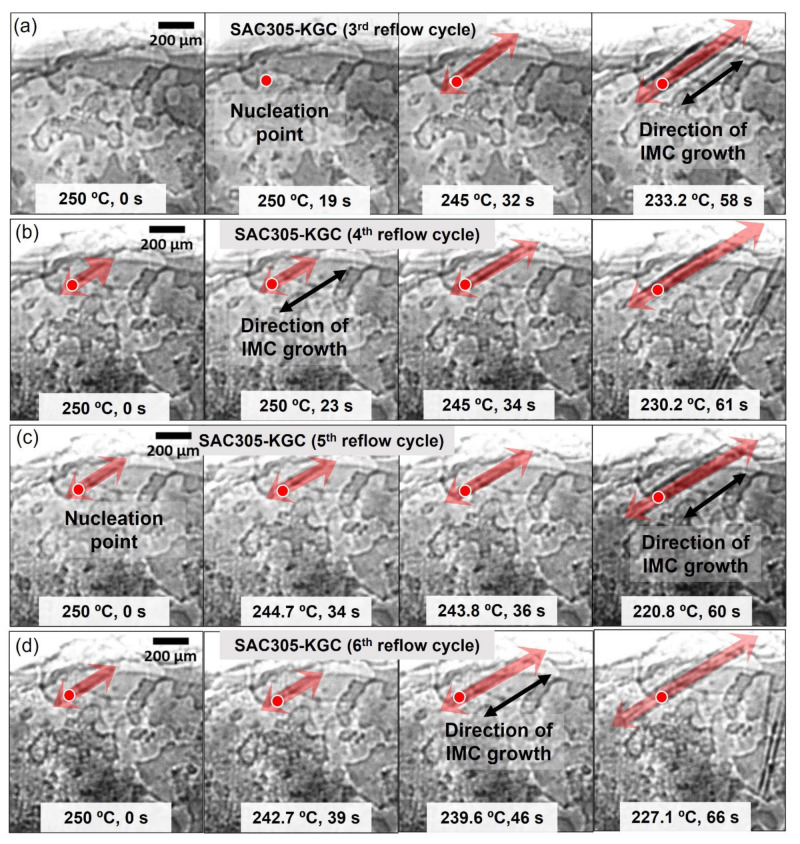
Sequences of the image showing the growth of IMC in SAC305-KGC solder joints during (**a**) 3rd reflow cycle, (**b**) 4th reflow cycle, (**c**) 5th reflow cycle, and (**d**) 6th reflow cycle (t = 0 s when the sample begins to cool).

It can be seen in Figure 6c–f that there is one primary Cu_6_Sn_5_ IMC (denoted with ‘g’) nucleated at the exact locations during the third to the sixth cycle of reflow soldering. Meanwhile, in the SAC305-KGC, most primary IMCs marked as ‘a’, ‘b’, ‘c’, ‘d’, ‘e’, and ‘f’ were observed to form at the same locations during the third to sixth cycle reflow soldering (per Figure 7c–f). To further elucidate the finding, snapshot images for primary Cu_6_Sn_5_ IMC (denoted by ‘g’) in SAC305 and one of the primaries in SAC305-KGC are shown in Figure 8 and Figure 9, respectively. Primary Cu_6_Sn_5_ IMC (denoted by ‘g’) was first nucleated at 227.6 °C, 50 s during the cooling from peak temperature in the third cycle reflow soldering (Figure 8a). Then, this primary IMC was fully melted during the heating process of the fourth reflow cycle and nucleated again at a similar location during the cooling at the following fifth and sixth reflow cycles. Additionally, this primary IMC has one growth direction, per Figure 8, during the multiple reflow cycle. This observation can be caused by the orientation indexed of the crystal structure. Cu_6_Sn_5_ IMC existed as a close-packed hexagonal crystal structure at a temperature above 186 °C with the orientations index of <0001> [11], and it can be inferred that the primary Cu_6_Sn_5_ IMCs in SAC305 will preferably grow in one growth direction. Meanwhile, in the SAC305-KGC, the primary Cu_6_Sn_5_ IMC was first nucleated after 19 s of cooling from a peak temperature of 250 °C, per Figure 9a. However, it should be pointed out that during the subsequent heating process in the fourth, fifth, and sixth cycles, the primary Cu_6_Sn_5_ IMC was not fully melted. However, during cooling, the primary Cu_6_Sn_5_ IMC will instantly grow in two growth directions until it solidifies, as depicted in Figure 9b–d. Generally, the fewer the crystal orientations of the IMC, the easier for the IMC to grow and increase in size due to the lower energy consumption during melting [11,36]. A key finding in this work is that (i) primary Cu_6_Sn_5_ IMC in SAC305-KGC nucleated earlier compared to SAC305, (ii) primary Cu_6_Sn_5_ IMC in SAC305-KGC do not fully melt during the heating stages of fourth, fifth, and sixth reflow cycle, and yet the primary IMCs will instantly grow during the cooling stages, and (iii) the additions of KGC in SAC305 causes the primary Cu_6_Sn_5_ IMCs growth in two growth directions. Despite that, the heating and cooling conditions (time and temperature) used during the soldering of both samples, SAC305 and SAC305-KGC, were similar. Therefore, this observation must be explained during the multiple reflow soldering of SAC305 and SAC305-KGC. Deep etching metallographic technique on the solder joints of SAC305 and SAC305-KGC was conducted to evaluate the possible effects of KGC addition on the growth of the primary Cu_6_Sn_5_ IMC.

**Figure 10:**
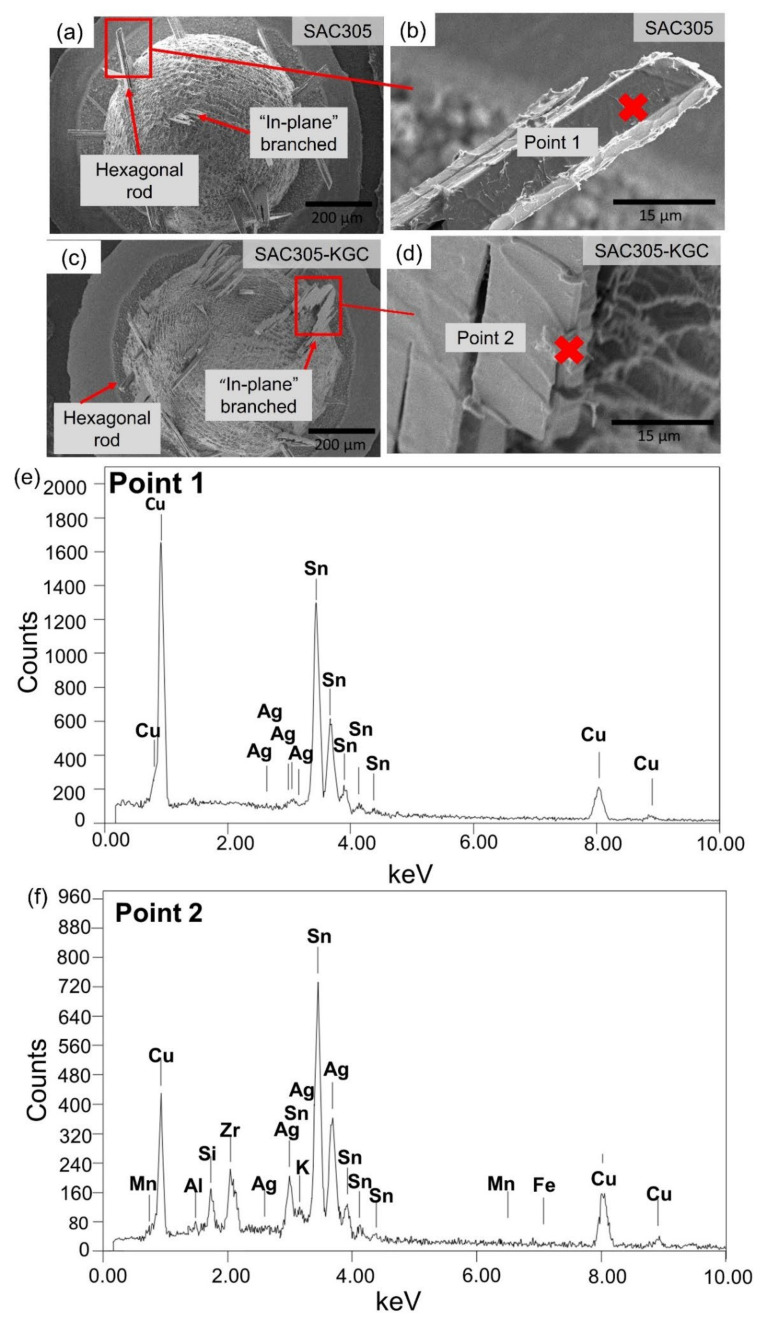
Top-down view of primary IMC in the bulk solder (**a**) Low magnification of SAC305, (**b**) Low magnification of SAC305-KGC, (**c**) High magnification of SAC305, (**d**) High magnification of SAC305-KGC. EDX point analysis at primary IMC in (**e**) SAC305, and (**f**) SAC305-KGC.

The deep etching technique removed tin and partially exposed primary Cu_6_Sn_5_ IMC_._ Figure 10 shows the top-down images for both the SAC305 and SAC305-KGC solder joints after the sixth cycle of reflow soldering. It can be seen that both solder joints consist of hexagonal rod and “in-plane” branched type of primary IMC, which aligned with the synchrotron radiation images, per Figure 6 and Figure 7. EDX analysis was performed on the primary Cu_6_Sn_5_ IMC in SAC305 and SAC305-KGC solder joints, and it was determined that the small agglomerations at the edge of primary Cu_6_Sn_5_ in SAC305-KGC are kaolin geopolymer ceramic (KGC) particles, as confirmed by EDX analysis results shown in Figure 10d. Al, Si, K, Mn, Fe, K, and Zr originated from the KGC systems. Based on this observation, it is possible that the KGC particles can be in contact with primary Cu_6_Sn_5_ IMC during multiple reflow soldering. It is hypothesized that KGC particles at the primary Cu_6_Sn_5_ IMC can explain the earlier nucleation in the SAC305-KGC solder joints. It can also be hypothesized that the presence of KGC particles can disturb the fully dissolved primary Cu_6_Sn_5_ IMC during the subsequent cycles of reflow soldering.

**Figure 11:**
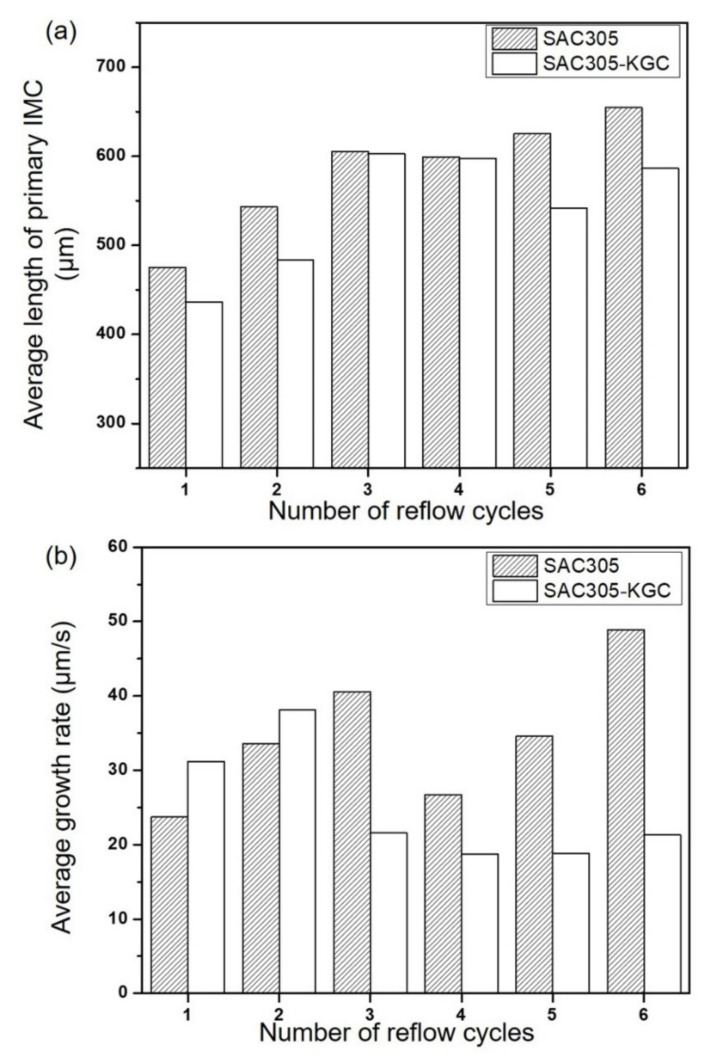
(**a**) Average length of the primary IMC in SAC305 and SAC305-KGC, and (**b**) Average growth rate of primary IMC in SAC305 and SAC305-KGC.

The growth behavior of primary Cu_6_Sn_5_ IMC at SAC305 and SAC305-KGC was quantified and shown in Figure 11. The final solidified primary Cu_6_Sn_5_ IMCs’ length in SAC305 and SAC305-KGC is shown in Figure 11. The length of the primary Cu_6_Sn_5_ was measured from its first nucleation until it was completely solidified. Figure 11b shows the growth rate of primary Cu_6_Sn_5_ for SAC305 and SAC305-KGC solder joints. Based on the graph in Figure 11a, the final solidified length of primary Cu_6_Sn_5_ in SAC305-KGC is relatively smaller than SAC305. The primary Cu_6_Sn_5_ in SAC305-KGC grew to a maximum average length of ~602 µm. In the case of SAC305, the primary Cu_6_Sn_5_ could grow to a maximum average length of ~ 654 µm. The differences in the maximum value of the final average solidified length of primary Cu_6_Sn_5_ in SAC305-KGC and SAC305 were ~8%. The long primary Cu_6_Sn_5_ in the solder joints compromised its reliability, as discussed in [36,37]. The differences in the size of primary IMCs can also be linked to the indexed orientations of the crystal structure [36]. As mentioned previously, the fewer growth orientations for IMCs, the easier for the IMCs to grow and increase in size due to their lower energy consumption. Similar to the case of the SAC305-KGC, the addition of KGC can slightly change the growth orientations of the primary Cu_6_Sn_5_, resulting in much smaller-sized primary IMCs. Additionally, in the case of the SAC305-KGC, the primary Cu_6_Sn_5_ IMC grew at the maximum average growth rate of ~38 µm/s, while in the case of SAC305, the maximum average growth rate of the primary Cu_6_Sn_5_ was ~49 µm/s. This led to the conclusion that the primary Cu_6_Sn_5_ IMC in SAC305-KGC grew to a shorter length at a slower rate. A key finding in this work is that the solidified length of primary Cu_6_Sn_5_ IMC in SAC305-KGC was relatively smaller, forming at a slower growth rate whilst experiencing earlier nucleation during multiple reflow soldering compared to SAC305. This can be attributed to the addition of KGC particles suppressing the growth of primary intermetallic. Salleh et al. [1] reported that the reinforcement particles in Sn-0.7Cu solder decreased the number density and total length per unit area of the primary Cu_6_Sn_5_ during multiple reflow soldering.

The results indicated that the suppression of intermetallic both as primary crystals and interfacial intermetallic layer is evident in materials of SAC305-KGC. As suggested by Gu et al. [24,38], the suppression of IMC was attributed to the ability of reinforcement particles to act as surface-active materials adsorbed onto solid surfaces. The reinforcement particles in the composite solder can be surface-active materials due to their high surface tension [38]. It is known that the smaller the size of the particles, the larger the surface tension and the specific surface area is. According to adsorption theory, the surface energy of Cu_6_Sn_5_ can be expressed as follows:

(3) $$\sum_{N}\gamma_{C}^{N}S_{N}=\sum_{N}\gamma_{O}^{N}S_{N}-RT\sum_{N}S_{N}\int_{O}^{C}\frac{\Pi^{N}}{c}\;dc\to \min$$

where *c* is the concentration of KGC particles, *γ^N^* is the surface tension of Cu_6_Sn_5_ particle *N*, $\gamma_{O}^{N}$ is the surface tension of Cu_6_Sn_5_ particle without adsorption of KGC, $\gamma_{C}^{N}$ is the surface tension of Cu_6_Sn_5_ particle with adsorption of KGC, $S_{N}$ is the area of Cu_6_Sn_5_ particle *N*, Π*^N^* is the number of KGC particles adsorbed by Cu_6_Sn_5_ particle *N*, *R* is gas constant, and *T* is the absolute temperature. From Equation (3), $\sum_{N}\gamma_{O}^{N}S_{N}$ is constant since it is not dependent on the concentration of KGC. Thus, the surface energy of Cu_6_Sn_5_ can be expressed as:

(4) $$\sum_{N}\gamma_{C}^{N}S_{N}=RT\;\sum_{N}S_{N}\;\int_{O}^{C}\frac{\Pi^{N}}{c}\;dc\to \max$$

Based on the relationship in Equation (4), it can be inferred that with an increasing amount of adsorbed KGC, the surface energy of Cu_6_Sn_5_ decreases. As indicated by the Gibs free energy, the decrease in the surface energy in Cu_6_Sn_5_ decreases the growth velocities of Cu_6_Sn_5_ and the growth rate for each of Cu_6_Sn_5_.

**Figure 12:**
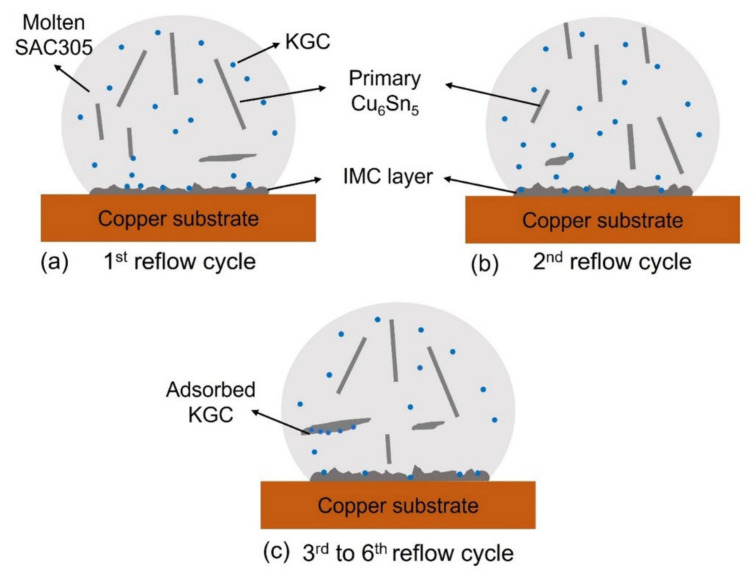
Propose mechanism on adsorption of KGC particles on the surface of Cu_6_Sn_5_ during multiple reflow soldering.

The proposed mechanisms are shown in Figure 12 for the effects of KGC particles, described as follows; during the reflow soldering, the KGC particles were likely to segregate into the molten solder [32]. As a result, some KGC particles were adsorbed on the primary Cu_6_Sn_5_ IMC and onto the copper substrate. Increasing the reflow cycle resulted in more KGC particles adsorbed onto the primary Cu_6_Sn_5_ IMC, which causes the growth rate of the primary Cu_6_Sn_5_ IMC to decrease from the third until the sixth cycle of reflow soldering, as depicted in Figure 11b, compared to SAC305. The growth orientations are likely to change with adsorbed KGC since primary Cu_6_Sn_5_ IMC exhibited two growth directions compared to SAC305, with one growth direction along with <0001> during multiple reflow soldering. Additionally, the adsorbed KGC is likely to disturb the melting of the primary Cu_6_Sn_5_ IMC during the subsequent heating cycle from the third to the sixth cycle of reflow soldering.

### 3.2. Shear Strength of Solder Joints after Multiple Reflows

**Figure 13:**
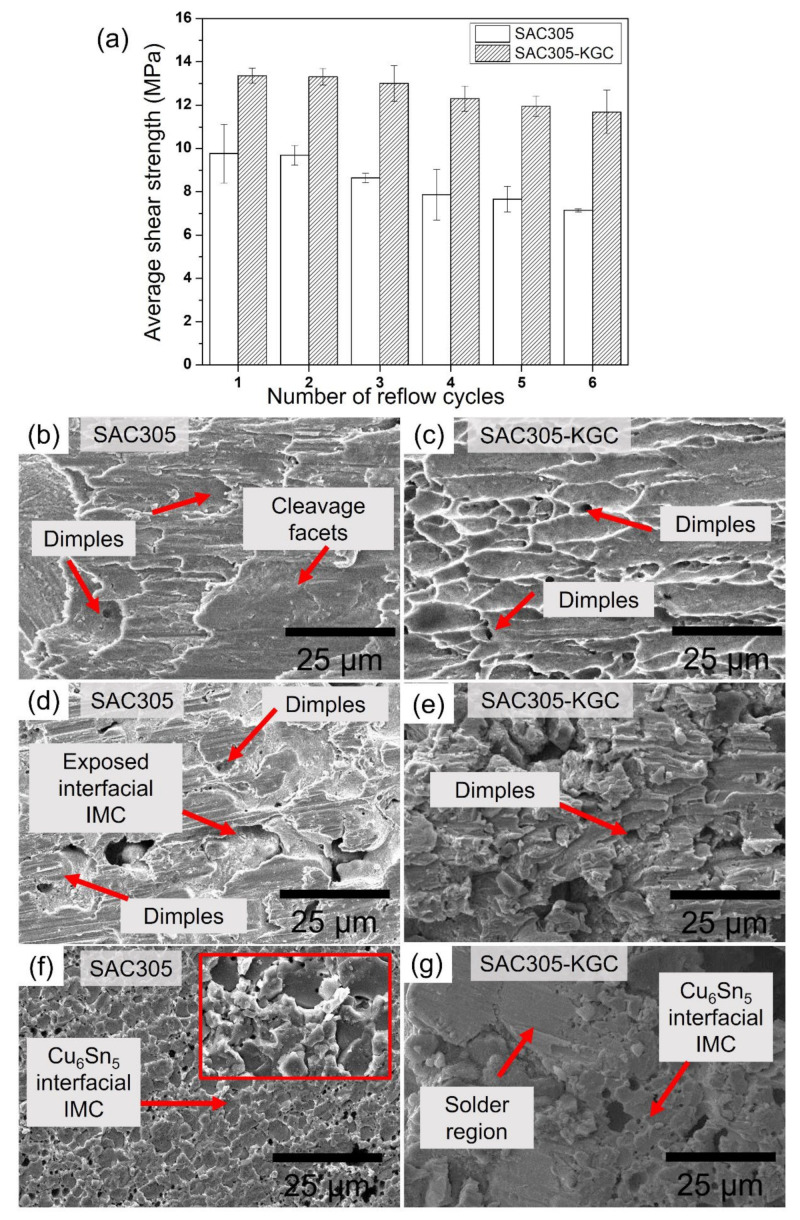
(**a**) Shear strength of SAC305 and SAC305-KGC subjected to multiple cycles of reflow soldering. SEM fracture surface of (**b**) SAC305 at 1st reflow, (**c**) SAC305-KGC at 1st reflow, (**d**) SAC305 at 3rd reflow, (**e**) SAC305-KGC at 3rd reflow, (**f**) SAC305 at 6th reflow, and (**g**) SAC305-KGC at 6th reflow cycle.

The mechanical performance of the solder joints was determined using a single lap shear test. Figure 13a shows the plot of the average shear strength for SAC305 and SAC305-KGC subjected to multiple cycles of reflow soldering. Overall, the average shear strength in SAC305 and SAC305-KGC decreased with increasing cycles of reflow soldering. However, the average shear strength in SAC305-KGC solder joints is higher than SAC305 regardless of the reflow soldering cycle. In the SAC305-KGC solder joints, the average shear strength showed ~13% reduction after the sixth cycle of reflow soldering compared with SAC305, which exhibited a 27% reduction after the sixth cycle of reflow soldering. A plausible explanation for the decrease in the average shear strength of the SAC305 is due to the formation of coarser microstructure and thicker interfacial IMC layer during the multiple cycles of reflow soldering. Meanwhile, the SAC305-KGC solder joints exhibited a lower reduction in the average shear strength after the sixth cycle of reflow soldering. This can be explained by the existence of KGC particles on both solder matrix and interfacial, as discussed in previous sections. The abovementioned results showed that the controllable coarsening in the microstructure and thinner interfacial IMC layer in SAC305-KGC benefitted the strength of solder during multiple cycles of reflow soldering. In addition, the relatively finer distribution of the IMCs in the solder bulk strengthened the solder matrix via dispersion strengthening [24]. Additionally, the smaller size of the primary IMCs in SAC305-KGC during multiple reflow soldering contributes to the strength of the solder joints.

A comprehensive analysis of the failure mechanism in SAC305 and SAC305-KGC involved using a scanning electron microscope (SEM) to elucidate the failure modes during multiple reflow soldering. Figure 13b–g shows the fractography for SAC305 and SAC305-KGC solder joints during the firth, third, and sixth reflow cycles. During the first reflow cycle, SAC305 solder joints failed in the combination of brittle and ductile failure mode. The appearance of shallow shear dimples was corresponding to the ductile region as in Figure 13b. The cleavage fracture area indicates less energy was absorbed during the shear test, which corresponds to the brittle region [43]. Meanwhile, SAC305-KGC solder joints show ductile fracture mode during the first cycle of reflow soldering with the appearance of shear dimples as in Figure 13c. Then, after the sixth cycle of reflow soldering, SAC305 solder joints showed a prominent structure of Cu_6_Sn_5_ IMC, suggesting that the failure occurred along with the IMC in a brittle manner after the shearing indicated in Figure 13f. On the other hand, in SAC305-KGC solder joints, a combined fractured mode (brittle and ductile) was observed after the sixth cycle of reflow soldering, per Figure 13g.

## 4. Conclusions

The effects of the addition of kaolin geopolymer ceramic in SAC305 solder joints were elucidated via the microstructural analyses at the bulk solder and the interfacial layer. The addition of KGC in the SAC305 solder suppressed the growth of the IMC both at the primary and interfacial layers and improved the shear strength of the solder. It can therefore be concluded that:

1. It was observed that the KGC particles remained in contact with the grains of the interfacial Cu_6_Sn_5_ IMC during multiple reflow soldering, which decreased the maximum average thickness of the IMC layer from ~12.6 µm (SAC305) to ~9.4 µm (SAC305-KGC). The scalloped interfacial IMC layer in SAC305-KGC became shorter and faceted after the sixth cycle of reflow. However, in SAC305, the elongated scalloped interfacial IMC layer grew longer into the solder’s matrix. The growth rate constant calculated for SAC305-KGC was 0.37 µm^2^/s, compared to SAC305, which is 0.81 µm^2^/s.
2. During the in situ microstructure analysis, the primary Cu_6_Sn_5_ IMC in SAC305-KGC nucleated earlier at higher temperatures during the cooling stage. As a result, the maximum average growth rate achieved in the SAC305-KGC was 38 µm/s compared to SAC305, which is 49 µm/s. The lower growth rate resulted in shorter lengths of solidified primary Cu_6_Sn_5_ IMCs in SAC305-KGC.
3. It was also observed that after the third cycle, the primary Cu_6_Sn_5_ IMCs in SAC305-KGC did not fully melt during subsequent heating of the fourth, fifth, and sixth cycle of reflow soldering and grew with two growth directions, which differs from SAC305 where the primary only grows with one growth direction. The results obtained were likely related to the mechanism of adsorption of KGC particles on the surface of primary Cu_6_Sn_5_ IMCs during multiple reflow soldering.
4. The suppression of Cu_6_Sn_5_ IMC both as primary and interfacial layers in SAC305-KGC resulted in a reduction of ~13% of average shear strength after multiple reflow soldering. However, in SAC305, the average shear strength decreased by ~27% after multiple reflows soldering and experiencing the brittle fracture mode.

## Data Availability

Data is contained within the article.
